# Skeletal versus conventional anchorage in dentofacial orthopedics: an international modified Delphi consensus study

**DOI:** 10.1186/s40510-025-00556-4

**Published:** 2025-03-03

**Authors:** Lorenzo Franchi, Maria Denisa Statie, Tommaso Clauser, Marco Migliorati, Alessandro Ugolini, Rosaria Bucci, Roberto Rongo, Riccardo Nucera, Marco Portelli, James A. McNamara, Michele Nieri, Sercan Akyalcin, Fernanda Angelieri, Daniele Cantarella, Paolo Cattaneo, Lucia Cevidanes, Luca Contardo, Marie Cornelis, Renzo De Gabriele, Carlos Flores Mir, Daniela Garib, Giorgio Iodice, Antonino Lo Giudice, Luca Lombardo, Björn Ludwig, Cesare Luzi, Maria Costanza Meazzini, Peter Ngan, Tung Nguyen, Alexandra Papadopoulou, Spyridon N. Papageorgiou, Jae Hyun Park, Sabine Ruf, Bernardo Souki, Benedict Wilmes, Heinz Winsauer

**Affiliations:** 1 Department of Experimental and Clinical Medicine, University of Florence, Florence, Italy; 2 Department of Sciences Integrated Surgical and Diagnostic, University of Genoa, Genoa, Italy; 3 Department of Neurosciences, Reproductive Sciences and Oral Sciences, University of Naples Federico II, Naples, Italy; 4 Department of Biomedical and Dental Sciences and Morphofunctional Imaging, University of Messina, Messina, Italy; 5 Department of Orthodontics and Pediatric Dentistry, The University of Michigan, Ann Arbor, USA; 6 Department of Developmental Biology, Harvard School of Dental Medicine, Boston, USA; 7 Private practice, Porto Feliz, São Paulo, Brazil; 8 Arizona School of Dentistry & Oral Health, A.T. Still University, Mesa, USA; 9 Melbourne Dental School, Faculty of Medicine, Dentistry and Health Sciences, University of Melbourne, Melbourne, Australia; 10 Department of Medicine, Surgery and Health Sciences, University of Trieste, Trieste, Italy; 11 Private practice, Lecce, Italy; 12 Division of Orthodontics, School of Dentistry, University of Alberta, Edmonton, Canada; 13 Bauru Dental School, Hospital for Rehabilitation of Craniofacial Anomalies, University of São Paulo, São Paulo, Brazil; 14 Department of General Surgery and Medical-Surgical Specialties, University of Catania, Catania, Italy; 15 Postgraduate School of Orthodontics, University of Ferrara, Ferrara, Italy; 16 Saarland University, Saarbrücken, Germany; 17 San Paolo Hospital, University of Milan and San Gerardo Hospital, Monza, Italy; 18 Department of Orthodontics, School of Dentistry, West Virginia University, Morgantown, USA; 19 School of Dentistry, University of North Carolina at Chapel Hill, Chapel Hill, USA; 20 Division of Orthodontics, University Clinics of Dental Medicine, Faculty of Medicine, University of Geneva, Geneva, Switzerland; 21 Clinic of Orthodontics and Pediatric Dentistry, Center for Dental Medicine, University of Zürich, Zürich, Switzerland; 22 Department of Orthodontics, University of Giessen, Giessen, Germany; 23 School of Dentistry, Graduate Program in Orthodontics, Pontifical Catholic University of Minas Gerais, Belo Horizonte, Brazil; 24 Department of Orthodontics, University of Düsseldorf, Düsseldorf, Germany; 25 Private practice, Bregenz, Austria; 26 Private practice, Rome, Italy

**Keywords:** Maxillary transverse deficiency, Class II malocclusion, Class III malocclusion, Skeletal anchorage

## Abstract

**Background:**

To establish consensus of skeletal anchorage versus conventional anchorage in treating: 1. Maxillary transverse deficiency in growing and adult patients, 2. Class II skeletal disharmony due to mandibular retrusion in growing patients, 3. Class III skeletal disharmony in growing patients.

**Methods:**

A four-rounds modified Delphi method was conducted. A steering committee performed a literature selection and compiled a list of 33 statements. An international panel of 25 experts in orthodontics agreed to participate. In each round, panelists were asked to rate their level of agreement with each statement using a 5-point Likert scale and provide comments. Statements that reached consensus were either accepted or rephrased. Statements that did not reach consensus were either rephrased, rejected, or split into two statements or merged with another.

**Results:**

After the four rounds, 24 statements achieved consensus while 9 were rejected. The distribution of consensus statements was as follows: Maxillary transverse deficiency: 4 statements; Class II skeletal disharmony: 10 statements; Class III skeletal disharmony: 10 statements.

**Conclusions:**

This modified Delphi consensus study aimed to provide guidance for orthodontists in choosing between skeletal and conventional anchorage for various treatment conditions. The study generated 24 consensus statements across three key domains. While the Delphi method provides valuable expert opinions, future studies, including randomized controlled trials, are needed to confirm these findings and address remaining uncertainties. Such efforts will aid in refining orthodontic treatment protocols and enhancing patient outcomes.

**Supplementary Information:**

The online version contains supplementary material available at 10.1186/s40510-025-00556-4.

## Introduction

Transverse maxillary deficiency, Class II, and Class III malocclusions are among the most common dentoskeletal imbalances encountered by orthodontists in daily practice [1]. Anchorage control is a fundamental principle in orthodontics, and for many years, orthodontists have sought methods of anchorage that minimize unwanted dental side effects and do not rely on patient cooperation. To address these challenges, skeletal anchorage has been increasingly integrated into orthodontic treatment over the past 40 years [2].

The ideal timing to treat maxillary transverse deficiency with conventional rapid maxillary expansion (RME) is before puberty [3]. Treatment of maxillary transverse deficiency through conventional RME in the pubertal and postpubertal stages can be challenging, as skeletal maturity and the increased interdigitation of the midpalatal suture make the orthopedic effect more difficult [3, 4]. This approach can lead to possible adverse effects at the periodontal level, particularly on the anchor teeth [5–7]. To minimize potential unwanted side effects, miniscrew-assisted rapid maxillary expansion (MARME) was developed to increase maxillary width in postpubertal and young adult patients [8, 9]. These benefits, however, come with the drawbacks of increased invasiveness and a higher risk of infection. [10, 11]

Class II skeletal disharmony is a common dentoskeletal imbalance that is characterized typically by mandibular retrusion [12]. Multiple removable and fixed functional appliances have been proposed during active growth in order to promote mandibular advancement [12]. Recently, to minimize dentoalveolar side effects and achieve greater skeletal effects, temporary anchorage devices (TADs) have been used in combination with fixed functional appliances (FFAs). Previous studies [13, 14] have demonstrated that FFA treatment, when reinforced with miniscrews or miniplates, reduces unwanted dentoalveolar effects, such as lower incisor proclination, and enhances the efficacy of orthopedic treatment in growing Class II patients. These studies [13, 14] reported favorable treatment outcomes particularly in terms of greater mandibular advancement, while other studies have disputed this effect [15].

Class III skeletal disharmony is considered typically one of the most challenging orthodontic problems for the practicing clinician [1]. RME combined with the facemask (FM) is one of the most effective treatment modalities for early intervention, as it stimulates intermaxillary and circummaxillary sutures, enhancing the orthopedic effect [16, 17]. However, tooth‐anchored RME/FM applies indirect orthopedic forces to the maxilla, causing side effects such as extrusion and mesial movement of the maxillary molars, proclination of the maxillary incisors, retroclination of the mandibular incisors, and backward and downward rotation of the mandible [17, 18]. Additionally, a key factor in achieving effective maxillary protraction and controlling mandibular growth is the strict age limit, with treatment being most effective in Class III patients under 11 years old and in the prepubertal stage [16, 19]. In order to overcome these potential limitations, the use of bone-anchored maxillary protraction protocols has been introduced to maximize skeletal effects and minimize dental effects [20, 21]. However, the major disadvantage of these protocols is the invasiveness involved in placing miniscrews or miniplates, along with the risk of postoperative inflammation, potential irritation of adjacent tissues, and failure of the skeletal anchorage due to insufficient bone quality at an early age [11, 22].

At present, there are many studies in the literature that provide controversial data and conclusions regarding the use of skeletal anchorage in the treatment of maxillary transverse deficiency, Class II, and Class III skeletal disharmony. Given the conflicting evidence regarding skeletal anchorage in the treatment of these malocclusions, an international modified Delphi study was conducted to establish an expert consensus on the use of skeletal versus conventional anchorage to treat maxillary transverse deficiency in growing and adult patients, Class II skeletal disharmony due to mandibular retrusion in growing patients, and Class III skeletal disharmony in growing patients.

## Materials and methods

### Chair, steering committee, and panelists

The present clinical consensus paper was reported according to ACCORD guidelines [23].

The study was not registered.

The consensus process was chaired by LF, who initially engaged two methodologists, MN and TC, to outline the subsequent steps. After identifying the main topic, three distinct subtopics were defined. Given the substantial differences among the subtopics, three research teams were formed, each consisting of two experts. To promote diversity, team members were selected from different research centers.

The steering committee consisted of:

- The chair (LF)
- Two methodologists (MN and TC)
- Research team for the treatment of maxillary transverse deficiency in growing patients and adult patients (MM and AU).
- Research team for the treatment of Class II skeletal disharmony due to mandibular retrusion in growing patients (RB and RR)
- Research team for the treatment of Class III skeletal disharmony in growing patients (RN and MP)
- A person who acted as a liaison between the steering committee and the panelists (MDS).

The steering committee conducted online meetings throughout various phases of the consensus process. During discussions, if members were unable to reach an agreement, the chair made the final decisions.

The chair selected 28 potential panelists based on their clinical experience and their contributions to research in the field of skeletal anchorage to join the panel of experts. There was no geographical constraint. The number of panelists was determined in agreement with the methodologists to ensure that consensus could be rejected only if more than two experts strongly disagreed. As a result, at least 21 participants were required, with a rejection rate of less than 25% expected.

Potential panelists were invited to participate via email by the chair. After receiving confirmation, 25 panelists were included in the panel. No funding or reimbursement was provided to any participant.

### Literature review and statements definition

Three literature searches were conducted by the research teams on their respective topics of interest. The search strings are listed in Table 1.

**Table 1:** Research strings for the topics of the consensus on Pubmed

Research group	Research string
Maxillary transverse deficiency	(Maxillary expansion,Palatal[MeSH Major Topic]) AND (orthodontic anchorage procedure[MeSH Terms] OR micro-implant OR microimplant OR miniscrew OR mini-screw OR "temporary anchorage device" OR "palatal implant" OR "midpalatal implant" OR mini-plate OR miniplate)) AND (hyrax OR haas OR marpe OR sarpe OR hybrid OR tandem OR orthopedic OR orthopedic OR growing OR child* OR adolescen* OR adult*)
Class II skeletal disharmony due to mandibular retrusion	((((Malocclusion, Angle Class II[MeSH Major Topic]) OR (retrognathism[MeSH Terms])) OR (("Class II" OR "Class 2"))) AND (orthodontic anchorage procedure[MeSH Terms] OR micro-implant OR microimplant OR miniscrew OR mini-screw OR "temporary anchorage device" OR "palatal implant" OR "midpalatal implant" OR mini-plate OR miniplate)) AND (functional OR interceptive OR orthopedic OR orthopaedic OR growing OR child* OR adolescen*)
Class III skeletal disharmony	(Bone-Anchored OR bone anchored or bone anchorage) OR (Skeletal anchorage OR temporary anchorage devices OR tads) OR (mini-screws OR mini screws OR miniscrews) OR (mini-implants OR mini implants OR miniimplants) OR (mini-plates OR miniplates OR mini plates) OR (micro-implant OR microimplant OR micro implant) OR Alt-RAMEC AND ("Malocclusion, Angle Class III"[Mesh]) OR (class III malocclusion) or (mandibular prognathism)

The research was performed on September 28th, 2023

The literature research was performed on PubMed, and each research team designed a research string according to their topic. The eligibility criteria were: Population (P): Growing or adolescent patients in the permanent dentition, or adult patients with maxillary transverse deficiency; growing or adolescent patients with Class II skeletal disharmony due to mandibular retrusion or with Class III skeletal disharmony. Intervention (I): Patients treated with bone-anchored maxillary expanders or with bone-anchored fixed or removable functional appliances or with bone-anchored Class III devices with or without Alternate Rapid Maxillay Expansion and Constriction (Alt-RAMEC) protocol. Comparison (C); Patients treated with tooth-anchored maxillary expanders, fixed or removable functional appliances and Class III devices with or without Alt-RAMEC protocol. Outcome (O): Dentoskeletal effects, complications. Study design (S): systematic reviews, randomized controlled trials, clinical controlled trials, case series.

The review had two primary aims:

1. To identify lack of evidence within their field of expertise that could be addressed in the consensus process.
2. To select approximately 10 papers that would be shared later with all the panelists, ensuring that the selected papers represented the highest level of evidence available for each topic (e.g. if a systematic review on a subject was found, randomized clinical trials were not included).

During this phase, the steering committee met several times to resolve any methodological uncertainties among members and to finalize the selection of papers to be shared with the panelists. After the literature selection, the three research teams compiled a list of potential statements, with about 10 items per team, though no strict limits were set due to the varying number of areas of interest that could emerge for each team's topic. This list was then discussed and refined in collaboration with the entire steering committee during a meeting.

Each research team defined a tentative list of statements, which were then discussed and refined by the entire steering committee during a meeting. The statements were intentionally kept brief and focused on a single topic to ensure that panelists’ feedback in the subsequent phases could be accurately interpreted. All statements and communications between panelists and the steering committee were conducted in English. A native English speaker (JAM) performed a technical language revision.

### Consensus rounds

A maximum of four rounds was established a priori.

Each round consisted of three phases: voting, consensus assessment, and statement decision.

#### Voting

Each panelist was assigned a randomly generated code using RStudio (RStudio Team, 2020). Specifically, a substring of the last four characters from randomly generated Universally Unique Identifiers (UUIDs) was checked for duplicates and then assigned to each participant. Only MDS had access to the code assignments throughout the consensus process. Members of the steering committee did not participate in the voting.

A Google Docs™ Forms document was created, requiring all panelists to enter their assigned code. No other identifiable information (e.g., name or email) was requested to ensure that the steering committee remained unaware of the panelists' identities when reviewing comments. For each statement, panelists were asked to select their level of agreement using a 5-point Likert scale (Table 2) and to provide comments to assist the steering committee in rephrasing the statement for the next phase. While selecting an agreement level was mandatory, leaving a comment was required only if the panelist chose 'neutral,' 'disagree,' or 'strongly disagree’.

**Table 2:** The Likert scale items and their meaning

Likert value	Meaning
1	Strongly disagree
2	Disagree
3	Neutral
4	Agree
5	Strongly agree

All panelists were asked to submit their responses by the specified due date, which ranged from 1 to 3 weeks depending on the magnitude of the expected work. If a panelist failed to respond by the deadline, MDS would identify the missing code and inform the chair, who would then remind the panelist of the due date. The anonymity of the panelists was preserved, as comments in the final reports were not ordered by submission date, nor was the date of submission disclosed. If a panelist failed to respond after three reminders, their name would be removed from the panelist list.

#### Consensus assessment

Once all panelist responses were collected, the round proceeded to the consensus assessment. A report was generated using RStudio, which included a bar chart for each statement, showing the number of panelists who selected each level of agreement. The report also indicated the consensus status (i.e., 'consensus' or 'no consensus') and included participant comments. Each comment was accompanied by the panelist’s code and their level of agreement for that statement. Consensus was considered achieved when the following three criteria were met simultaneously:

- At least 70% of the panelists 'agreed' or 'strongly agreed' with the statement.
- Fewer than 20% of the panelists 'disagreed' or 'strongly disagreed.'
- Fewer than 10% of the panelists 'strongly disagreed' with the statement.

The criteria were defined a priori and were not changed during the consensus development.

The report was then shared with the steering committee and with the panelists.

#### Statement decision

Each team independently reviewed the results and formulated a tentative decision for each statement. These decisions were then discussed with the entire steering committee for confirmation or revision. In cases where the steering committee could not reach a consensus, it was planned that the chair would make the final decision. However, this situation did not occur.

A statement that reached consensus was either:

1. **Accepted**: An accepted statement would not be included in the next round of voting since consensus had already been achieved; or
2. **Rephrased**: Based on the panelists' comments, the statement was rephrased to potentially gain broader consensus in the steering committee's view. It would then be included in the next round of voting. If, in subsequent rounds, the statement failed to achieve consensus or gained lower consensus, the version that achieved the highest consensus would be accepted.

A statement that did not reach consensus was:

1. **Rephrased**: Similarly, the statement could be rephrased to aim for wider consensus in the next round;
2. **Rejected**: If the steering committee determined that consensus could not be reached due to differing opinions among panelists, the statement would be excluded from future rounds; or
3. **Split into two statements or merged with another**: If the steering committee believed a statement might achieve wider consensus if split or combined with another, it would be adjusted accordingly for the next round. This option was avoided, when possible, to maintain clarity.

After the steering committee finalized the decisions on each statement, a new form was prepared so that rephrased, split, or merged statements could be voted on. After the fourth round, statements could only be accepted or rejected.

## Results

The first meeting of the steering committee was held on July 14th, 2023. The selection of evidence was planned to be completed and sent by the end of January. A few update meetings were scheduled for September 28th, 2023, October 26th, 2023, and November 23rd, 2023.

Meanwhile, the chair sent invitations to 28 potential panelists. Twenty-five individuals accepted the invitation and were subsequently included in the panel (Table 3).

**Table 3:** Panelists included in the Delphi study

Name of the panelist	Affiliation
Sercan Akyalcin	Harvard School of Dental Medicine, Boston, Massachusetts, USA
Fernanda Angelieri	Private practice, Porto Feliz, Sâo Paulo, Brazil
Daniele Cantarella	ATSU, Arizona School of Dentistry & Oral Health, Mesa, Arizona, USA
Paolo Cattaneo	University of Melbourne, Melbourne, Australia
Lucia Cevidanes	University of Michigan, Ann Arbor, Michigan, USA
Luca Contardo	University of Trieste, Trieste, Italy
Marie Cornelis	University of Melbourne, Melbourne, Australia
Renzo De Gabriele	Private practice, Lecce, Italy
Carlos Flores Mir	University of Alberta, Edmonton, Alberta, Canada
Daniela Garib	University of São Paulo, Bauru, São Paulo, Brazil
Giorgio Iodice	University of Naples Federico II, Naples, Italy
Antonino Lo Giudice	University of Catania, Catania, Italy
Luca Lombardo	University of Ferrara, Ferrara, Italy
Björn Ludwig	Homburg University, Homburg, Germany
Cesare Luzi	Private Practice, Rome, Italy
Maria Costanza Meazzini	San Paolo Hospital, University of Milan and S.Gerardo Hospital, Monza, Italy
Peter Ngan	West Virginia University, Morgantown, West Virginia, USA
Tung Nguyen	University of North Carolina, Chapel Hill, North Carolina, USA
Alexandra Papadopoulou	University of Geneva, Geneva, Switzerland
Spyridon Papageorgiou	University of Zurich, Zurich, Switzerland
Jae Hyun Park	ATSU, Arizona School of Dentistry & Oral Health, Mesa, Arizona, USA
Sabine Ruf	University of Giessen, Giessen, Germany
Bernardo Souki	Pontifical Catholic University of Minas Gerais, Belo Horizonte, Minas Gerais, Brazil
Benedict Wilmes	University of Dusseldorf, Dusseldorf, Germany
Heinz Winsauer	Private practice, Bregenz, Austria

On January 29th, 2024, the final selection of 12 papers was confirmed during a meeting (Supplementary Table) [8–11, 13, 14, 20, 21, 24–27].

In the same meeting, a list of topics for the statements was also established. The papers were sent to the panelists on February 7th, 2024.

On February 22nd, 2024, the steering committee met to finalize the list of statements. A total of 32 statements were defined: 9 on treatment of maxillary transverse deficiency, 12 on treatment of Class II skeletal disharmony, and 11 on treatment of Class III skeletal disharmony.

### First round

Following the English language revision, the first round of consensus began on March 14th, 2024, and the voting phase concluded on April 5th. Response rate of the panelists was 100%. Out of 32 statements, 9 reached consensus: 2 on the treatment of maxillary transverse deficiency, 3 on the treatment of Class II skeletal disharmony, and 4 on the treatment of Class III skeletal disharmony. On April 12th, 2024, the steering committee met to define a new list of statements. Of the 9 statements that reached consensus, 4 were accepted as they were, and 5 were rephrased. Among the 23 statements that did not reach consensus, 21 were rephrased, 1 was split and 1 was rejected. As a result, 28 statements advanced to the second round: 8 on the treatment of maxillary transverse deficiency, 10 on the treatment of Class II skeletal disharmony, and 10 on the treatment of Class III skeletal disharmony.

### Second round

The second round began on April 16th, 2024, with a due date of May 1st. After follow-up reminders were sent to late panelists, the voting phase was extended and closed on May 14th. Response rate of the panelists was 100%. Fifteen statements reached consensus, while 13 did not. On May 23, 2024, the steering committee met to finalize a new list of statements. All 15 statements that reached consensus were accepted. Of the 13 statements that did not reach consensus, 4 were rejected and 9 were rephrased. Consequently, 9 statements advanced to the third round.

### Third round

The third round began on June 3rd, 2024, with a due date set for June 18th. The voting phase closed on July 5th, following reminders to late panelists. Response rate of the panelists was 100%. Of the 9 statements, 4 reached consensus. On July 12th, 2024, the steering committee met to finalize the new list of statements. The 4 statements that reached consensus were accepted. Of the remaining 5 statements, 4 were rejected and 1 was rephrased.

### Fourth round

The fourth round began on June 21, 2024, with a due date of June 26. After reminders were sent to late panelists, the voting phase closed on August 5, 2024. Response rate of the panelists was 100%. The only remaining statement reached consensus and was subsequently accepted. In total, consensus was achieved on 24 out of the 32 statements. The final statements are presented in Table 4 (treatment of maxillary transverse deficiency in growing and adult patients), Table 5 (treatment of Class II skeletal disharmony due to mandibular retrusion in growing patients), and Table 6 (treatment of Class III skeletal disharmony in growing patients).

**Table 4:** Results on treatment of maxillary transverse deficiency

#	Statement	Strongly disagree	disagree	Agree and strongly agree	Consensus	Last round of appearance
	**Treatment of maxillary transverse deficiency in growing patients in the permanent dentition**					
1	Bone-anchored expanders produce a greater skeletal expansion (opening of the midpalatal suture) when compared to tooth-anchored expanders, although this effect is relatively small and probably not clinically relevant	4%	20%	72%	No	2nd
2	Bone-borne expanders reduce the buccal inclination of expanded posterior teeth compared to tooth-anchored expanders	0%	0%	100%	Yes	1st
3	Bone-anchored expanders reduce the risk of developing gingival recessions on permanent molars when compared to tooth-anchored expanders	0%	8%	52%	No	1st
4	Bone-anchored expanders do not produce more stable medium- or long-term results when compared to tooth-anchored expanders, although the level of evidence is low	4%	24%	64%	No	3rd
	**Treatment of maxillary transverse deficiency in late adolescents and adult patients**					
5a	Bone-anchored expanders are effective in producing maxillary skeletal expansion in late adolescent patients	0%	0%	96%	Yes	2nd
5b	Bone-anchored expanders are effective in producing maxillary skeletal expansion in adult patients with the success rate decreasing with age	0%	12%	72%	Yes	2nd
6	When using bone-anchored expanders, it is not possible to predict the opening of midpalatal suture, particularly in patients older than 25–30 years	4%	4%	88%	Yes	2nd
7	Based on clinical experience, slow activation protocol seems to improve the results of bone-anchored expanders	0%	16%	48%	No	2nd
8	The characteristics of the bone-anchored expanders (digital planning, number and dimensions of the miniscrews, connection arms between expansion screw and miniscrews, etc.) can affect the results of expansion, although the level of evidence is low	0%	20%	72%	No	3rd
9	Gingival inflammation and pain are rare side effects during and after active expansion with bone-anchored expanders	0%	28%	72%	No	3rd

**Table 5:** Results on treatment of Class II skeletal disharmony due to mandibular retrusion in growing patients

#	Statement	Strongly disagree	Disagree	Agree and strongly agree	Consensus	Last round of appearance
1	Fixed functional appliances with skeletal anchorage in the lower jaw produce a greater sagittal skeletal correction compared to tooth-anchored fixed functional appliances, although the level of evidence is low	0%	16%	76%	Yes	3rd
2	Fixed functional appliances with skeletal anchorage in the lower jaw do not produce a greater sagittal skeletal correction compared to removable functional appliances, although the level of evidence is low	0%	28%	60%	No	3rd
3	Fixed functional appliances with skeletal anchorage in the lower jaw provide greater control of lower incisors proclination compared to tooth-anchored fixed functional appliances	0%	4%	88%	Yes	1st
4	Fixed functional appliances with skeletal anchorage in the lower jaw provide greater control of lower incisor proclination compared to removable functional appliances	0%	8%	72%	Yes	1st
5	Fixed functional appliances with skeletal anchorage in the lower jaw do not provide greater control of mandibular clockwise rotation compared to tooth-anchored fixed functional appliances	0%	4%	84%	Yes	2nd
6	Fixed functional appliances with skeletal anchorage in the lower jaw do not provide greater control of mandibular clockwise rotation compared to removable functional orthopedic appliances	0%	0%	80%	Yes	2nd
7	The use of fixed functional appliances anchored to miniscrews in the upper and lower jaws is preferable to the use of fixed functional appliances anchored to miniscrews in the lower jaw only, when retraction of the upper arch is not indicated	4%	20%	64%	No	2nd
8	Considering the less invasiveness, fixed functional appliances anchored to miniscrews are preferable to fixed functional appliances anchored to miniplates	0%	8%	88%	Yes	2nd
9	Patient’s acceptance of bone-anchored fixed functional appliances is more challenging compared to tooth-anchored devices	4%	8%	76%	Yes	3rd
10	In cases with specific indications (i.e. lower incisors proclination), the benefits of bone-anchored fixed functional appliances justify the higher biological risks (e.g. surgical procedure and post-surgical recovery) compared to tooth-anchored fixed functional appliances	4%	0%	88%	Yes	3rd
11	In cases with specific indications (i.e. lower incisors proclination), the benefits of bone-anchored fixed functional appliances justify the higher costs compared to tooth-anchored fixed functional appliances	4%	0%	84%	Yes	3rd
12	Bone-anchored fixed functional appliances are indicated to control lower incisor proclination	4%	0%	88%	Yes	2nd

**Table 6:** Results on treatment of Class III skeletal disharmony in growing patients

#	Statement	Strongly disagree	Disagree	Agree and strongly agree	Consensus	Last round of appearance
1	Bone-anchored devices produce a greater sagittal skeletal correction compared to tooth-anchored appliances, although this effect is relatively small and probably not clinically relevant, particularly in prepubertal patients	0%	12%	84%	Yes	2nd
2	Bone-anchored devices are more effective in reducing the dentoalveolar effects compared to tooth-anchored appliances	0%	0%	100%	Yes	1st
3	Bone-anchored devices reduce the counterclockwise rotation of the palatal plane observed with tooth-anchored appliances and facemask, although this effect is relatively small and probably not clinically relevant	0%	8%	88%	Yes	2nd
4	Bone-anchored devices reduce the clockwise rotation of the mandibular plane observed with tooth-anchored appliances and facemask, although this effect is relatively small and probably not clinically relevant	0%	12%	80%	Yes	2nd
5	Bone-anchored Alt-RAMEC protocol is more effective compared to tooth-anchored Alt-RAMEC protocol, particularly in pubertal patients, although the evidence is low	0%	12%	72%	Yes	4th
6	Biological invasiveness of bone-anchored devices is justified by their greater efficacy compared to tooth-anchored devices, in pubertal patients	0%	16%	84%	Yes	2nd
7	The economic impact of bone-anchored devices is justified by their greater efficacy compared to tooth-anchored devices, in pubertal patients	0%	16%	80%	Yes	2nd
8	The increased invasiveness and complications of surgical procedures of BAMP protocol are not justified by the potential benefit of improved compliance due to a fully intraoral device	0%	48%	44%	No	3rd
9	Failure rate of bone-anchored orthopedic devices with miniplates is higher in patients younger than 11 years	0%	4%	88%	Yes	2nd
10	When bone-anchored orthopedic devices emerge through non-keratinized tissue there is a greater chance of inflammation and infection that can increase the risk of failure	0%	8%	88%	Yes	2nd
11	The failure rate of bone-anchored orthopedic devices is higher when miniscrews are positioned in interradicular sites rather than in palatal and mandibular buccal shelf sites	0%	12%	88%	Yes	2nd

### Consensus

#### *Treatment of maxillary transverse deficiency (*Table 4*)*

Among 4 statements on treating maxillary transverse deficiency in growing patients with permanent dentition, experts reached consensus on only one. They agreed that bone-borne expanders reduce the buccal inclination of expanded posterior teeth compared to tooth-anchored expanders.

Regarding the treatment of maxillary transverse deficiency in late adolescent and adult patients, experts found consensus for 3 out of 6 statements, agreeing that bone-anchored expanders are effective in producing maxillary skeletal expansion in both late adolescent and adult patients with the success rate decreasing with age. Moreover, they considered not predictable the opening of the midpalatal suture, particularly in patients older than 25–30 years.

#### *Treatment of Class II skeletal disharmony due to mandibular retrusion in growing patients (*Table 5*)*

Of the 12 statements regarding the treatment of Class II skeletal disharmony due to mandibular retrusion in growing patients, experts reached consensus on 10. They agreed that fixed functional appliances with skeletal anchorage in the lower jaw produce a greater sagittal effect than tooth-anchored fixed functional appliances, although the supporting evidence is limited. Experts agreed that bone-anchored fixed functional appliances effectively control lower incisor proclination. In specific cases, such as those involving lower incisor proclination, the higher biological risks and costs associated with bone-anchored fixed functional appliances are considered justified over those of tooth-anchored appliances. However, patient acceptance of bone-anchored appliances can be more challenging than with tooth-anchored devices.

Additionally, experts concluded that bone-anchored fixed functional appliances in the lower jaw do not offer greater control of mandibular clockwise rotation compared to tooth-anchored fixed functional appliances or removable functional orthopedic appliances. Furthermore, due to their less invasive nature, fixed functional appliances anchored to miniscrews are preferred over those anchored to miniplates.

#### *Treatment of Class III skeletal disharmony in growing patients (*Table 6*)*

Out of 11 statements on treatment of Class III skeletal disharmony in growing patients, experts found consensus for 10 statements. Experts agreed that bone-anchored devices produce greater sagittal skeletal correction compared to tooth-anchored appliances. Bone-anchored devices also reduce the counterclockwise rotation of the palatal plane and the clockwise rotation of the mandibular plane typically seen with tooth-anchored appliances and FM. However, both these effects are relatively small and likely not clinically significant. On the other hand, experts agreed that bone-anchored devices are more effective in reducing dentoalveolar effects compared to tooth-anchored appliances. Additionally, bone anchored Alt-RAMEC protocol is more effective compared to tooth-anchored Alt-RAMEC protocol, particularly in pubertal patients, although the evidence is low.

Regarding invasiveness and failure rate, experts agreed that in pubertal patients the high biological invasiveness and the high economic impact of bone-anchored devices are justified by their greater efficacy compared to tooth-anchored devices. However, in children younger than 11 years the failure rate of the miniplates is higher. When bone-anchored orthopedic devices emerge through non-keratinized tissue there is a greater chance of inflammation and infection that can increase the risk of failure. Finally, the failure rate of bone-anchored orthopedic devices is higher when miniscrews are positioned in interradicular sites rather than in palatal and mandibular buccal shelf sites.

## Discussion

The literature presents conflicting opinions regarding the use of temporary titanium screws or plates (TADs) to achieve desired skeletal modifications while minimizing unwanted dental side effects. The aim of this international modified Delphi study was to establish an expert consensus on the use of skeletal versus conventional anchorage in growing and adult patients with maxillary transverse deficiency, Class II skeletal disharmony in growing patients, and Class III skeletal disharmony in growing patients. The panelists consisted of 25 international experts from various countries, who participated in four rounds, during which statements on these topics were proposed.

### Treatment of maxillary transverse deficiency

#### Treatment of maxillary transverse deficiency in growing patients in the permanent dentition

Regarding growing patients in the permanent dentition, no consensus was reached among the panelists on statement #1 (Table 4): Bone-anchored expanders produce a greater skeletal expansion with opening of the midpalatal suture compared to tooth-anchored expanders, although this effect is relatively small and probably not clinically relevant. However, 72% of the panelists agreed with this statement. This statement was supported by the results of Krusi et al. [9] who found limited evidence from randomized trials indicating that bone-borne or hybrid tooth-bone-borne expanders might offer advantages in terms of increased sutural opening compared to conventional tooth-borne expanders. Similarly, Bi and Li [8] concluded that MARME (miniscrew-assisted rapid maxillary expansion) resulted in greater skeletal transverse expansion compared to conventional RME. However, while this result was statistically significant, it was not considered clinically relevant.

As for the dentoalveolar effects, 100% of the panelists agreed that, in growing patients, bone-borne expanders reduce the buccal inclination of expanded posterior teeth compared to tooth-anchored expanders (statement #2, Table 4). Krusi et al. [9] concluded that both bone-borne and hybrid expanders were associated with less buccal tipping of the first premolar compared to tooth-borne expanders, although the meta-analysis showed a moderate to high risk of bias in the RCTs. Some panelists noted that while fewer undesirable dental effects may be expected, more evidence is still needed.

Consensus was not reached for statement #3 (Table 4) as only 52% of the panelists agreed that bone-anchored expanders reduce the risk of developing gingival recessions on permanent molars when compared to tooth-anchored expanders. The lack of agreement was due to the insufficient evidence regarding this outcome. Although Bi and Li [8] showed that MARME can reduce the buccal inclination of the maxillary first premolars and first molars, the correlation between buccal inclination of the upper posterior teeth and gingival recession is weak and does not directly reflect the assessment of recessions. In addition, Ventura et al. [28] concluded that there is limited evidence on the impact of MARME on the periodontium, whose changes are considered clinically insignificant and comparable to RME.

Even though consensus was not reached, 64% of the panelists agreed that bone-anchored expanders do not provide more stable medium- or long-term results compared to tooth-anchored expanders (statement #4, Table 4). Most panelists highlighted the low level of evidence, as trials with short follow-ups do not show significant differences between the two types of expanders. For this reason, it was emphasized that more studies evaluating the medium- and long-term stability of bone-anchored versus tooth-anchored expanders are necessary.

#### Treatment of maxillary transverse deficiency in late adolescent and adult patients

A substantial number of experts, 96% and 72%, agreed that bone-anchored expanders are effective in producing maxillary skeletal expansion in late adolescent and adult patients (though the success rate decreases with age), respectively (statements #5a and #5b, Table 4). The panelists highlighted that, for patients older than 18 years old, the success rate decreases with age [10]. In line with this, consensus was achieved on statement #6 (Table 4): “When using bone-anchored expanders it is not possible to predict the opening of the midpalatal suture, particularly in patients older than 25–30 years” [29, 30].

Regarding the slow activation protocol (Statement #7, Table 4 “Based on clinical experience slow activation protocol seems to improve the results of bone-anchored expanders”), no consensus was obtained, with only 48% of panelists agreeing or strongly agreeing with its effectiveness.

The statement #8 “The characteristics of the bone-anchored expanders (digital planning, number and dimensions of the miniscrews, connection arms between expansion screw and miniscrews, etc.) can affect the results of expansion, although the level of evidence is low” (Table 4) caused much controversy among the panelists. A majority of the panelists (72%) agreed that these factors could influence the outcome of the expansion, as the size of TADs and bicorticalism have been related to their capacity to support load and stress [31]. On the other hand, a recent study showed bicortical anchorage was significantly better in the posterior maxilla, while there was no difference in failure rate on monocortical vs bicortical screws in the anterior maxilla [32]. However, 20% of the panelists disagreed with this statement preventing consensus.

As for the side effects during and after active expansion with bone-anchored expanders in late adolescents and adult patients, Yoon et al. [10] found that the most frequent complication was inflammation (83.9%), and 45% of patients reported pain or tenderness during expansion. However, there was no consensus among the panelists, with 28% disagreeing with these findings (statement #9, Table 4). Some experts emphasized that, if the appliance is properly designed, pain or inflammation should not occur during active expansion. In contrast, others stressed that pain is expected in late adolescent and adult patients.

### Treatment of Class II skeletal disharmony due to mandibular retrusion in growing patients

Panelists agreed that fixed functional appliances with skeletal anchorage in the lower jaw produce a greater sagittal skeletal correction and provide greater control of lower incisors proclination compared to tooth-anchored fixed functional appliances (statements #1 and #3, Table 5). These consensus were supported by a meta-analysis [14], that showed that compared to FFA (Fixed Functional Appliance) alone, patients treated with FFA combined with TADs demonstrated significant differences in SNB angle, Co-Gn length, ANB angle, and lower incisor–mandibular plane angle (LI-MP). These results indicated that skeletally anchored FFA can promote mandibular advancement, enhance mandibular growth, and reduce lower incisor inclination. This meta-analysis [14] showed moderate to high risk of bias with a high heterogeneity in the designs of the selected studies. Although consensus was reached, it was noted that the level of evidence is low. Additionally, some panelists emphasized that while mandibular changes are statistically significant, they may not be clinically relevant.

No consensus was reached on statement #2 (Table 5) that proposed that FFAs with skeletal anchorage in the lower jaw do not produce greater sagittal skeletal correction compared to removable functional appliances. The lack of agreement was due to the poor evidence in the literature, as highlighted by the panelists. Ince-Bingol et al. [25], who compared the treatment efficacy of the activator and skeletally anchored Forsus appliance, found no significant difference between the two approaches in terms of mandibular advancement.

Consensus was reached among the panelists on statement #4 (Table 5), that stated that FFAs with skeletal anchorage in the lower jaw provide greater control of lower incisor proclination compared to removable functional appliances. In particular, Ince-Bingol et al. [25] concluded that the lower incisor retroclination achieved with the skeletally anchored Forsus appliance provided more advantageous and stable results, as removable functional appliances tended to cause lower incisor proclination, that can be an undesirable side effect.

Regarding mandibular vertical control, panelists agreed that FFAs with skeletal anchorage in the lower jaw do not offer greater control of mandibular clockwise rotation compared to either fixed or removable functional appliances (statements #5 and #6, Table 5). They also suggested that further studies are needed to clarify this issue. The meta-analysis by Huang et al. [14] revealed that, compared to conventional FFAs, patients treated with FFAs anchored to miniscrews showed no significant difference in the change of the SN-MP angle.

No consensus was reached on Statement #7 (Table 5), that proposed that using FFAs anchored to miniscrews in both the upper and lower jaws is preferable to using them in the lower jaw only when retraction of the upper arch is not indicated. This lack of consensus was due to insufficient evidence in the literature on this topic.

The panelists agreed that, given the less invasive nature, FFAs anchored to miniscrews are preferable to those anchored to miniplates (statement #8, Table 5). Although the meta-analysis by Huang et al. [14] demonstrated greater mandibular skeletal effects with FFAs anchored to miniplates compared to miniscrews, Cubuk et al. [11] found that miniplates were significantly more invasive and had a notable failure rate (mobility and breakage) of around 12%. Nevertheless, some panelists noted that in cases where larger skeletal changes are desired, miniplates may be warranted to provide more reliable anchorage than miniscrews when applying orthopedic forces.

Since patients' acceptance of orthodontic appliances and their level of compliance can impact their satisfaction with treatment outcomes [33], panelists were asked to assess patients’ experiences during orthodontic procedures. They agreed that patients’ acceptance of bone-anchored FFAs is more challenging compared to tooth-anchored devices (statement #9, Table 5). Elkordy et al. [13], however, showed that the insertion of mini-implants did not cause significant discomfort during appliance placement, and no significant differences in patient experiences were found when comparing bone-anchored vs conventional Forsus appliance. Unexpectedly, there were no significant differences regarding tooth pain, cheek irritation and swelling during appliance therapy between the groups, even though the patients treated with conventional Forsus appliance responded slightly better than did the patients treated with the bone-anchored Forsus appliance.

Consensus was achieved in concluding that bone-anchored FFAs are indicated to control lower incisor proclination, which justifies the higher biological risks (e.g., surgical procedures and post-surgical recovery) and costs compared to tooth-anchored FFAs (statements #10–12, Table 5).

### Treatment of Class III skeletal disharmony in growing patients

In the present Delphi study, a consensus was reached among the panelists that bone-anchored devices produce a greater sagittal skeletal correction compared to tooth-anchored appliances, although this effect is relatively small and probably not clinically relevant, particularly in prepubertal patients (statement #1, Table 6). Regarding ANB angle and Wits appraisal, a recent systematic review [27] showed that there were no statistical differences in either measurements between treatment of Class III patients with a dentally-anchored FM versus a skeletally-anchored FM. Furthermore, Rutili et al.[27] concluded that differences in SNA angle between the two groups were statistically significant but clinically negligible. Similar findings were reported by Wang et al. [21] in a systematic review with network meta-analysis. The authors found that bone anchored devices produced a greater maxillary protraction (SNA) and sagittal skeletal correction (ANB) when compared to conventional FM, although this effect was relatively small and probably not clinically relevant.

All panelists agreed that bone-anchored devices are more effective in reducing the dentoalveolar effects compared to tooth-anchored appliances (statement #2, Table 6). This statement agrees with the findings reported by recent systematic reviews [21, 22, 27]. Specifically, Rutili et al. [27] concluded that the inclination of the upper incisors relative to the cranial base (U1-SN angle) was significantly smaller at the end of treatment in the miniscrew group compared with the no miniscrew group.

Regarding vertical changes, there was consensus that bone-anchored devices reduce the counterclockwise rotation of the palatal plane and the clockwise rotation of the mandibular plane observed with tooth-anchored appliances and FM, although this effect is relatively small and probably not clinically relevant (statements #3 and #4, Table 6). Both Wu et al. [20] and Wang et al. [21] concluded that bone‐anchored approaches produced better vertical control than conventional RME and FM therapy. However, Rutili et al [27] showed a lack of differences in SN-MP angle between the miniscrew and no miniscrew groups.

Consensus among panelists was reached that bone-anchored Alt-RAMEC protocol is more effective compared to tooth-anchored Alt-RAMEC protocol, particularly in pubertal patients, although the evidence is low (statement #5, Table 6). The RCT performed by Alzoubi et al. [34] did not find any statistically significant differences between tooth borne versus skeletally anchored Alt-RAMEC protocol in prepubertal Class III patients. The retrospective study performed by Maino et al. [35] on the long-term effects produced by a skeletally-anchored Alt-RAMEC protocol in patients with an average initial age of 11 years, showed promising amount of maxillary advancement (SNA = + 2.5°) and change in ANB (+ 1.7°). In this study, however, no control sample was available.

Panelists agreed that in pubertal patients, biological invasiveness and economic impact of bone-anchored devices is justified by their greater efficacy compared to tooth-anchored devices (statements #6 and #7, Table 6). Most panelists noted that the use of bone-anchored devices is not recommended during the age range when the FM protocol has a high success rate (before puberty), but it can be considered when the circum-maxillary sutures have matured (at puberty). Additionally, they emphasized the importance of considering the type of skeletal anchorage, as miniscrews are less invasive than miniplates.

No consensus was reached on statement #8 (Table 6). Fourty-eight percent of the panelists disagreed that the increased invasiveness and complications of the BAMP protocol are not justified by the potential benefit of improved compliance due to the fully intraoral nature of the device. A few panelists argued that the favorable skeletal effects produced by the BAMP protocol justify the increased invasiveness and complications of the surgical procedures. Additionally, they noted that the reduced need for orthognathic surgery following the BAMP protocol makes the increased invasiveness worthwhile. Other panelists highlighted that the BAMP protocol with Class III elastics is more comfortable, leading to greater patient compliance compared to the FM. It was emphasized also that more data are needed on patient compliance with Class III elastics compared to the FM.

A consensus was reached among the panelists that the failure rate of bone-anchored orthopedic devices with miniplates is higher in patients younger than 11 years (statement #9, Table 6). This statement agrees with the findings of Van Hevele et al. [26], who reported a higher failure rate of miniplates in patients younger than 11 years (29.3%), although the difference was not statistically significant compared to the failure rate in patients older than 11 years (21.1%). Panelists pointed out that more evidence is required.

As for side effects, the panelists agreed that when bone-anchored orthopedic devices emerge through non-keratinized tissue there is a greater chance of inflammation and infection that can increase the risk of failure (statement #10, Table 6). The study by Van Hevele et al. [26] confirmed that placing the neck of the bone anchor in the attached gingiva resulted in lower failure rates. Although consensus on this statement was reached, some panelists noted that there is insufficient evidence to support this outcome, and further investigations are needed.

Finally, regarding the insertion site, the panelists agreed that there is a higher failure rate when miniscrews are positioned in interradicular sites compared to palatal and mandibular buccal shelf sites. Additionally, they emphasized that the clinical success of skeletal anchorage depends on the quality and quantity of bone at the miniscrew placement site.

### Clinical relevance

Experts generally agreed that bone-anchored devices produce fewer dentoalveolar effects compared to tooth-anchored devices. Specifically, bone-anchored expanders help minimize the buccal inclination of posterior teeth, skeletally anchored fixed functional appliances provide greater control over lower incisor proclination in the treatment of Class II skeletal disharmony, and bone-anchored devices are more effective at reducing dentoalveolar effects in the treatment of Class III skeletal disharmony.

Additionally, bone-anchored devices appear to produce greater sagittal skeletal effects than tooth-anchored devices in treating both Class II and Class III skeletal disharmonies. However, the evidence supporting this observation is limited, and the clinical significance of these effects may be minimal.

### Limitations and future recommendations

A critical aspect of a Delphi study is the selection of participants. For this consensus, participants were chosen by the chair based on his knowledge of the relevant literature and recommendations from the steering committee. This approach aimed to ensure a balanced representation of world-renowned researchers and expert practitioners.

Consensus statements are particularly valuable in areas where robust clinical evidence is limited. They provide interim guidance by synthesizing the collective expertise of specialists and the best available knowledge at the time. However, these statements are not a substitute for high-quality clinical evidence. As new and definitive evidence emerges, it is expected to refine and expand our understanding of the topic.

## Conclusions

This international modified Delphi consensus study aimed to provide guidance for orthodontists in choosing between skeletal and conventional anchorage for various treatment conditions. The study generated 24 consensus statements on the use of skeletal anchorage versus conventional anchorage in dentofacial orthopedic treatment, across three key domains.

While the Delphi method offers valuable expert opinions, future studies, including randomized controlled trials, are needed to confirm these findings and address remaining uncertainties. These efforts will contribute to refining orthodontic treatment protocols and improving patient outcome.

## Supplementary Information

Additional file1

## Data Availability

No datasets were generated or analysed during the current study.
