# Correction to: Skeletal versus conventional anchorage in dentofacial orthopedics: an international modified Delphi consensus study

**DOI:** 10.1186/s40510-025-00579-x

**Published:** 2025-09-19

**Authors:** Lorenzo Franchi, Maria Denisa Statie, Tommaso Clauser, Marco Migliorati, Alessandro Ugolini, Rosaria Bucci, Roberto Rongo, Riccardo Nucera, Marco Portelli, James A. McNamara, Michele Nieri, Sercan Akyalcin, Fernanda Angelieri, Daniele Cantarella, Paolo Cattaneo, Lucia Cevidanes, Luca Contardo, Marie Cornelis, Renzo De Gabriele, Carlos Flores Mir, Daniela Garib, Giorgio Iodice, Antonino Lo Giudice, Luca Lombardo, Björn Ludwig, Cesare Luzi, Maria Costanza Meazzini, Peter Ngan, Tung Nguyen, Alexandra Papadopoulou, Spyridon N. Papageorgiou, Jae Hyun Park, Sabine Ruf, Bernardo Souki, Benedict Wilmes, Heinz Winsauer

**Affiliations:** 1 Department of Experimental and Clinical Medicine, University of Florence, Florence, Italy; 2 Department of Sciences Integrated Surgical and Diagnostic, University of Genoa, Genoa, Italy; 3 Department of Neurosciences, Reproductive Sciences and Oral Sciences, University of Naples Federico II, Naples, Italy; 4 Department of Biomedical and Dental Sciences and Morphofunctional Imaging, University of Messina, Messina, Italy; 5 Department of Orthodontics and Pediatric Dentistry, The University of Michigan, Ann Arbor, USA; 6 Department of Developmental Biology, Harvard School of Dental Medicine, Boston, USA; 7 Private Practicr, Porto Feliz, São Paulo, Brazil; 8 Arizona School of Dentistry and Oral Health, A.T. Still University, Mesa, USA; 9 Melbourne Dental School, Faculty of Medicine, Dentistry and Health Sciences, University of Melbourne, Melbourne, Australia; 10 Department of Medicine, Surgery and Health Sciences, University of Trieste, Trieste, Italy; 11 Private Practice, Lecce, Italy; 12 Division of Orthodontics, School of Dentistry, University of Alberta, Edmonton, Canada; 13 Hospital for Rehabilitation of Craniofacial Anomalies, Bauru Dental School, University of São Paulo, São Paulo, Brazil; 14 Department of General Surgery and Medical‑Surgical Specialties, University of Catania, Catania, Italy; 15 Postgraduate School of Orthodontics, University of Ferrara, Ferrara, Italy; 16 Saarland University, Saarbrücken, Germany; 17 San Paolo Hospital, University of Milan and San Gerardo Hospital, Monza, Italy; 18 Department of Orthodontics, School of Dentistry, West Virginia University, Morgantown, USA; 19 School of Dentistry, University of North Carolina at Chapel Hill, Chapel Hill, USA; 20 Division of Orthodontics, Faculty of Medicine, University Clinics of Dental Medicine, University of Geneva, Geneva, Switzerland; 21 Clinic of Orthodontics and Pediatric Dentistry, Center for Dental Medicine, University of Zürich, Zürich, Switzerland; 22 Department of Orthodontics, University of Giessen, Giessen, Germany; 23 School of Dentistry, Graduate Program in Orthodontics, Pontifical Catholic University of Minas Gerais, Belo Horizonte, Brazil; 24 Departmentof Orthodontics, University of Düsseldorf, Düsseldorf, Germany; 25 PrivatePractice, Bregenz, Austria; 26 Private Practice, Rome, Italy

**Correction: Progress in Orthodontics (2025) 26:9** 10.1186/s40510-025-00556-4.

Following publication of the original article [1], the authors identified an error in the author name of Spyridon N. Papageorgiou.

The incorrect author name is: Spyridon Papageorgiou.

The correct author name is: Spyridon N. Papageorgiou.

The author group has been updated above and the original article [1] has been corrected.
