# Supplementary material for: Skeletal versus conventional anchorage in dentofacial orthopedics: an international modified Delphi consensus study
Source: Prog Orthod. 2025 Mar 3;26:9. doi: 10.1186/s40510-025-00556-4 (PMC11872959; doi:10.1186/s40510-025-00556-4)
Supplement: Supplementary file 1 — Additional file1 [file 40510_2025_556_MOESM1_ESM.docx]

Supplementary Table 1. List of papers provided to the panelists.

| Study | Topic | Type of study | Aim |
| --- | --- | --- | --- |
| Bi & Li 2022^8^ | Transverse | Systematic review | To compare the effectiveness and side effects of miniscrew-assisted rapid maxillary expansion (MARME) with conventional RME in the treatment of transverse maxillary deficiency. |
| Krüsi et al. 2019^9^ | Transverse | Systematic review | To compare the clinical effects of bone-borne or hybrid  tooth-bone-borne RME with conventional tooth-borne RME in the treatment of maxillary deficiency. |
| Yoon et al. 2022^10^ | Transverse | Case series | To identify the complications associated with miniscrew-assisted RME and the frequency of their occurrences. |
| Elkordy et al. 2016^13^ | Class II | Systematic review | To compare the skeletal and dentoalveolar effects of the use of fixed functional appliances (FFAs) with and without skeletal anchorage (miniscrews or miniplates). |
| Huang et al. 2021^14^ | Class II | Systematic review | To investigate the skeletal and dentoalveolar effects of FFAs with temporary anchorage devices on Class II malocclusion in adolescents. |
| Ince-Bingol et al. 2021^25^ | Class II | CCT | To investigate the treatment efficiency of miniplate anchored Forsus Fatigue Resistant Device as compared with the activator appliance. |
| Manni et al. 2019^24^ | Class II | CCT | To compare the efficacy of skeletally anchored Herbst treatment (with maxillary and mandibular miniscrews) versus conventional Herbst treatment for Class II malocclusion. |
| Rutili et al. 2023^27^ | Class III | Systematic review | To compare the dentoskeletal effects produced by the facemask with or without skeletal anchorage for the treatment of Class III malocclusion in growing patients. |
| Wang et al. 2022^21^ | Class III | Systematic review | To estimate the clinical effects of different types of bone-anchored maxillary protraction devices by using a network meta-analysis. |
| Wu et al. 2020^20^ | Class III | Systematic review | To evaluate and compare the effectiveness of orthopedic treatment for Class III malocclusions using skeletal anchorage or a rapid maxillary expander for maxillary protraction. |
| Van Hevele et al. 2018^26^ | Class III | Case series | To evaluate the impact of Class III correction by elastic traction on four miniplates and the failure rate of bone-anchored miniplates. |
| Çubuk et al. 2019^11^ | Class II and Class III | Case series | To evaluate success rates and complications related with symphyseal miniplate anchorage systems used for treatment of Class II and Class III deformities. |

CCT: Controlled Clinical Trial
